# Unusual Presentation of Primary Hyper-IgE-Related Salivary Gland Disease in a 13-Year-Old Male

**DOI:** 10.7759/cureus.48086

**Published:** 2023-10-31

**Authors:** Alden S Jason, Sneha Pendem, Murugesan Krishnan, Santhosh P Kumar

**Affiliations:** 1 Oral and Maxillofacial Surgery, Saveetha Dental College and Hospitals, Saveetha Institute of Medical and Technical Sciences, Saveetha University, Chennai, IND

**Keywords:** surgical excision, innovative technique, autoimmune sialadenosis, sialadenitis, salivary gland pathology

## Abstract

Adenomas are common pathologies of the salivary glands that are often associated with the major salivary glands and occur in the fourth to sixth decades of life. They are seldom seen in the pediatric age group and rarely in the minor salivary glands. Autoimmune sialadenosis of the minor salivary glands is a new phenomenon that has seldom been reported in the literature, with as few as three cases. Histopathological examination of the excised specimen is the definitive diagnosis, and these lesions have to be differentiated from adenomas and low-grade malignancies of the minor salivary glands. Management strategies of these lesions are extremely variable, ranging from wait-and-watch principle to the use of immunosuppressants and excision of the gland. This case report discusses the etiopathogenesis of the autoimmune sialadenosis and the management strategies.

## Introduction

Benign salivary gland lesions can manifest as either sialadenitis or sialadenosis. Sialadenitis can be of either an acute viral infectious nature or a more chronic inflammation as a result of obstruction or stricture [1]. On the other hand, sialadenosis typically presents as a painless, non-inflammatory bilateral swelling of the salivary glands associated with systemic conditions [2]. Among children, the most common salivary gland pathology is viral sialadenitis, commonly known as mumps, which primarily affects the parotid gland, although rare cases of submandibular salivary gland involvement have been reported in the literature [3].

Reports of sialadenosis have seldom been reported in the literature with most of them occurring in major salivary glands and in adults. However posterior palatal swellings originating from minor salivary glands can be of varied etiology like infections, autoimmune conditions, and neoplasms [1]. Adults presenting with such swellings are extremely common compared to the pediatric counterparts, necessitating a careful thorough evaluation. Autoimmune sialadenosis is a rare condition in children, and a meticulous evaluation is crucial to rule out neoplastic changes that can significantly impact the patient's quality of life [3]. This report describes a rare case of autoimmune immunoglobulin E (IgE) associated with sialadenosis of the minor salivary gland in a child and provides a detailed discussion on its possible etiopathogenesis and management strategies.

## Case presentation

**Figure 1:**
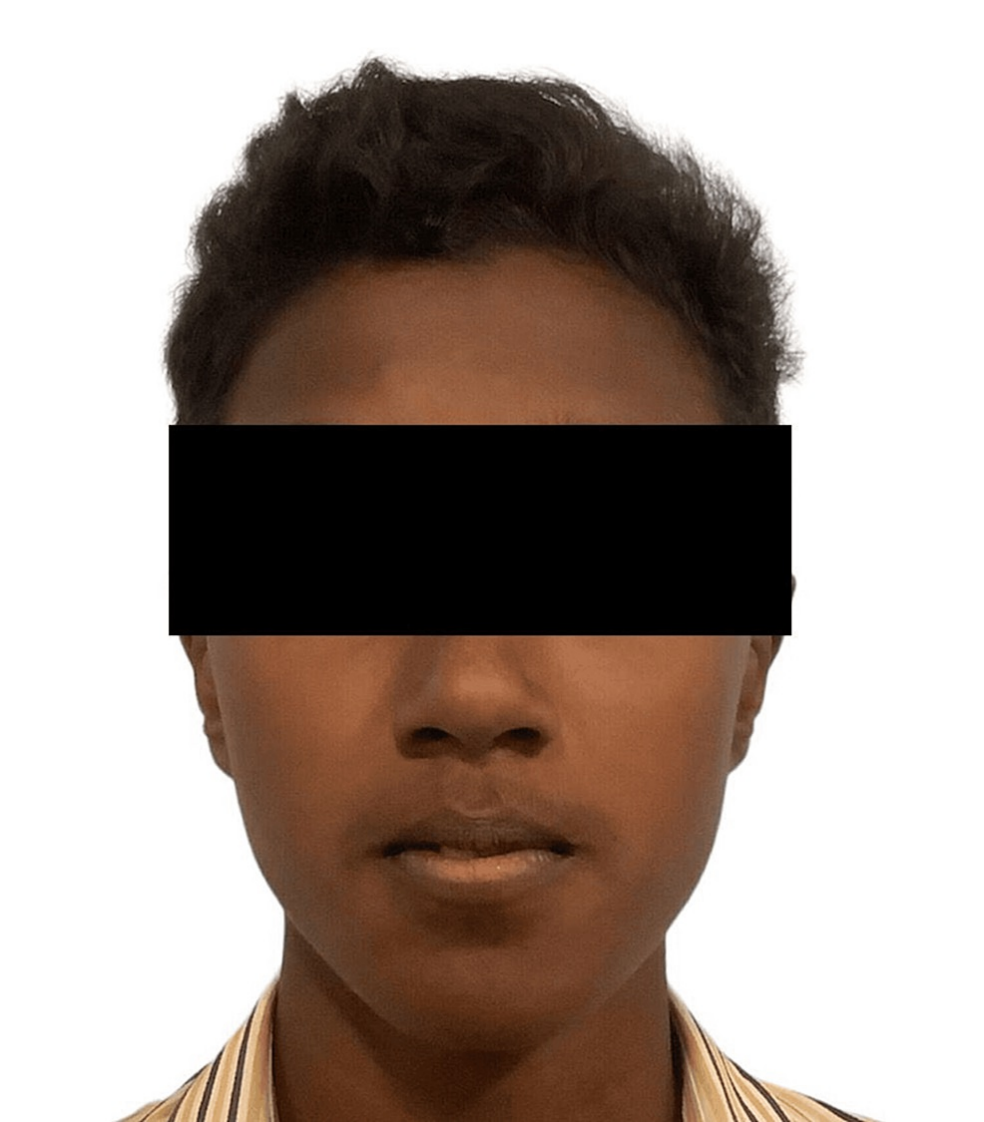
Extraoral picture of the patient

A 13-year-old male patient with fair systemic health and no known medical comorbidities presented to us with a complaint of swelling in the right posterior palate extending from first molar to third molar region (Figure 1). The patient's history revealed that the swelling had been present for the past six months with no concomitant discomfort, burning sensation, or discharge. The onset of the palatal swelling was insidious, and it had progressively increased to the current size. The patient experienced occasional difficulty in swallowing.

**Figure 2:**
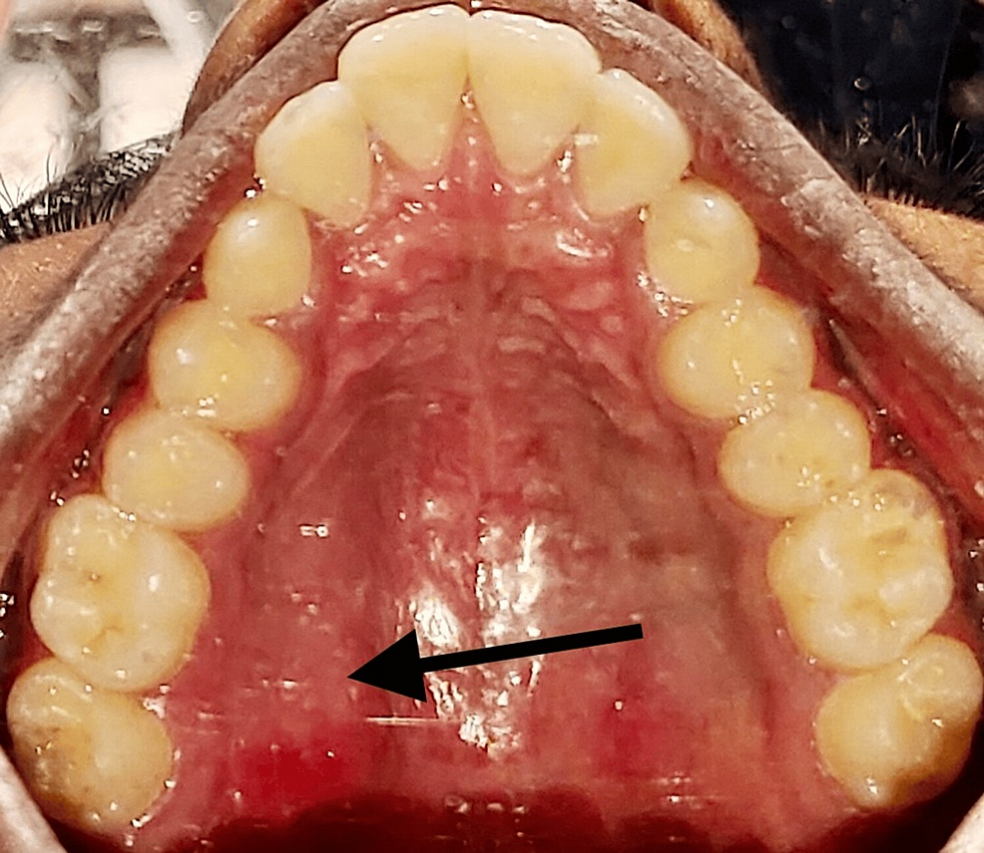
Intraoral image of the patient showing lesion in the palatal region (arrow)

**Figure 3:**
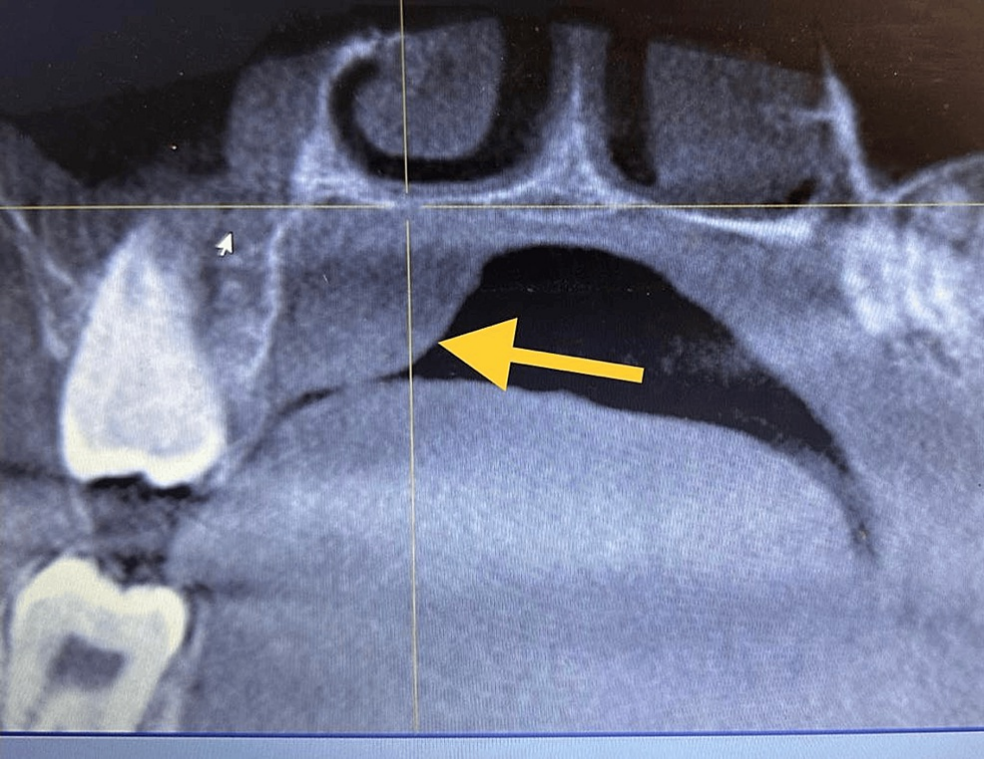
Coronal section of computed tomography showing uniform hypodense lesion with no erosion of the palatal bone (arrow)

Upon clinical examination, the swelling was well circumscribed and was about 4 × 3 cm in size. It was non-tender, non-compressible, and non-reducible, and was firm in consistency (Figure 2). The mucosa over the swelling was normal with no bleeding or discharge. Aspiration yielded negative results suggestive of a solid tumor, and the incisional biopsy report was inconclusive. Based on the findings, a provisional diagnosis of adenoma of the palatal salivary gland was arrived at. Cone beam computed tomography scan revealed the lesion to be uniformly hypodense with no erosion of the palatal bone overlying the greater palatine foramen (Figure 3) favoring the diagnosis of adenoma, and the patient was planned for wide local excision of the lesion.

**Figure 4:**
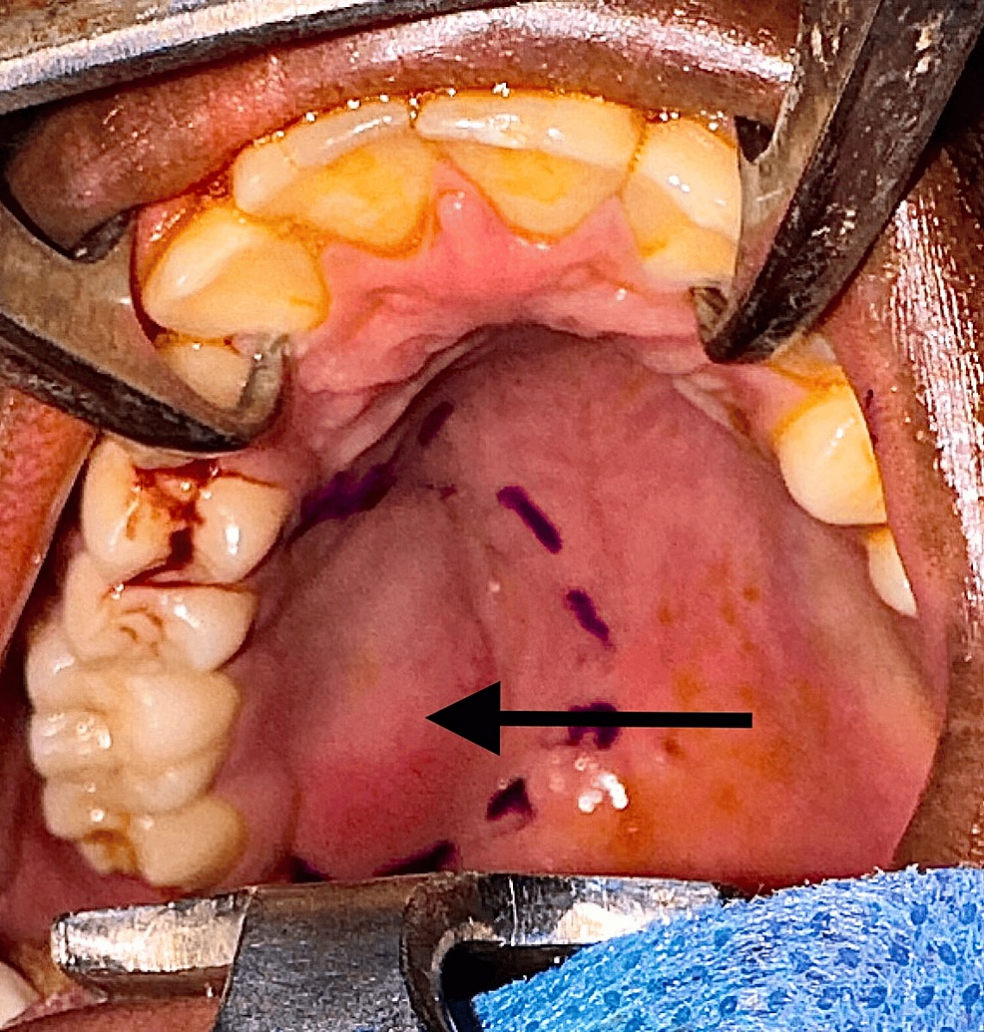
Markings for excision of the lesion placed (arrow)

**Figure 5:**
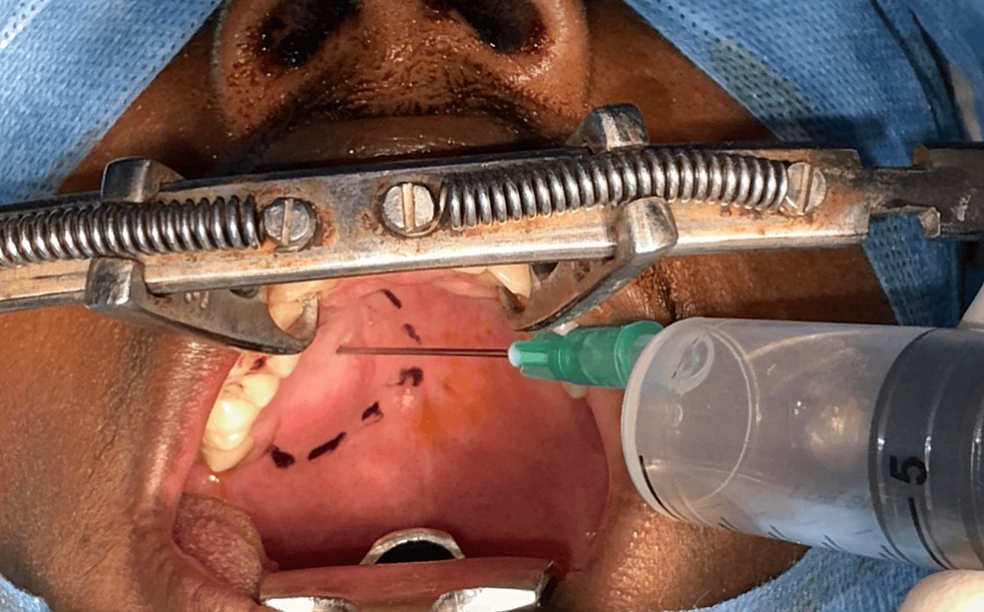
Aspiration of the lesion

Under general anesthesia, incision markings were done for clearance of one centimeter around the lesion (Figure 4). Aspiration of the lesion yielded negative results (Figure 5).

**Figure 6:**
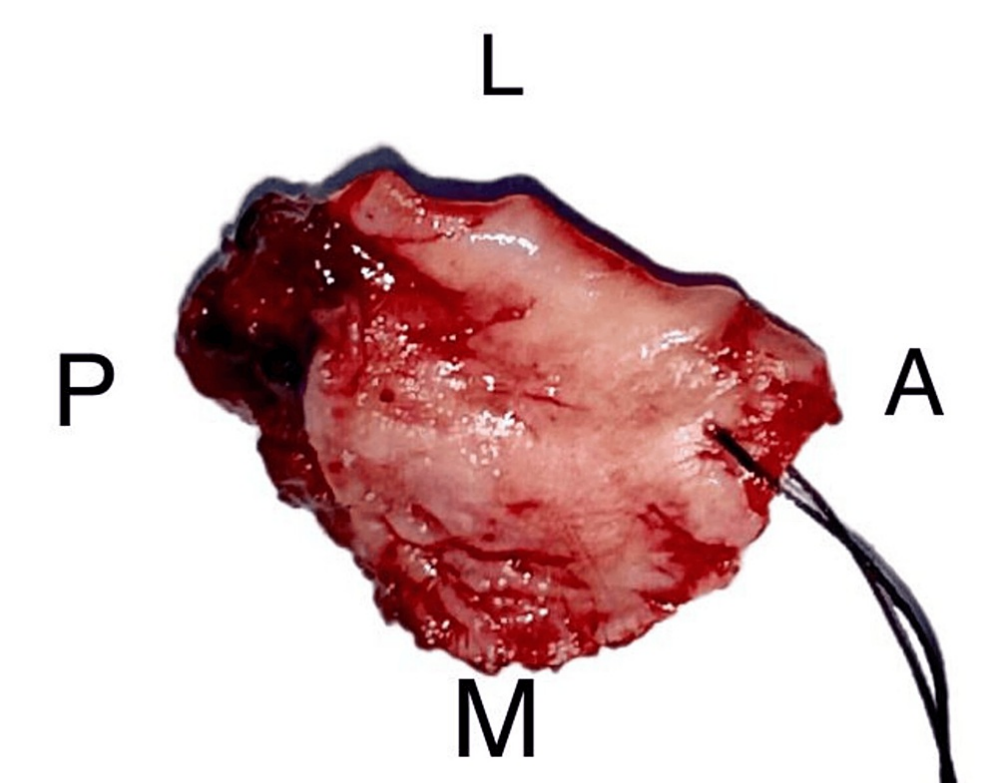
Excised specimen L: Lateral Surface; A: Anterior Surface; M: Mesial Surface; P: Posterior Surface

**Figure 7:**
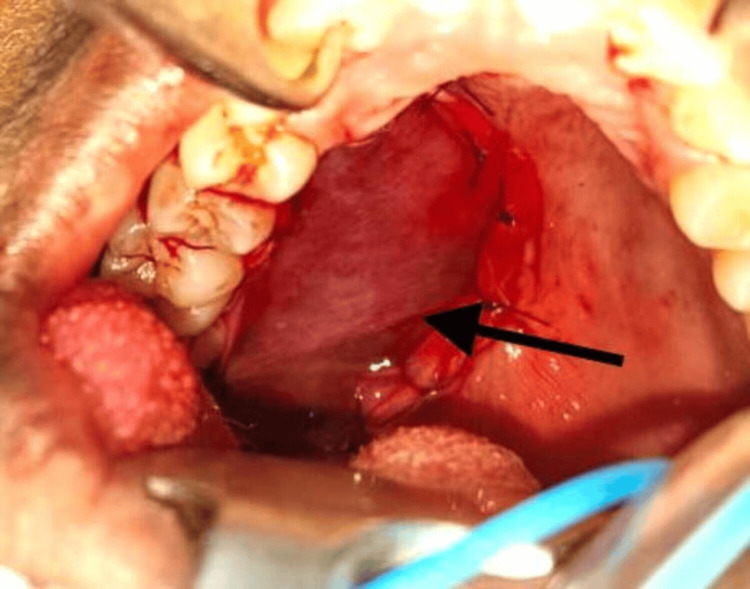
Surgical defect covered with collagen membrane and sutured (arrow)

The lesion was excised in toto with a circumferential margin of one centimeter (Figure 6). Greater and lesser palatine vessels were identified and cauterized to achieve hemostasis. Peripheral ostectomy of the palatal bone was carried out to achieve three-dimensional clearance. A collagen membrane was placed and secured over the exposed bone to achieve mucosal cover (Figure 7). The excised lesion was subjected to histopathological evaluation.

**Figure 8:**
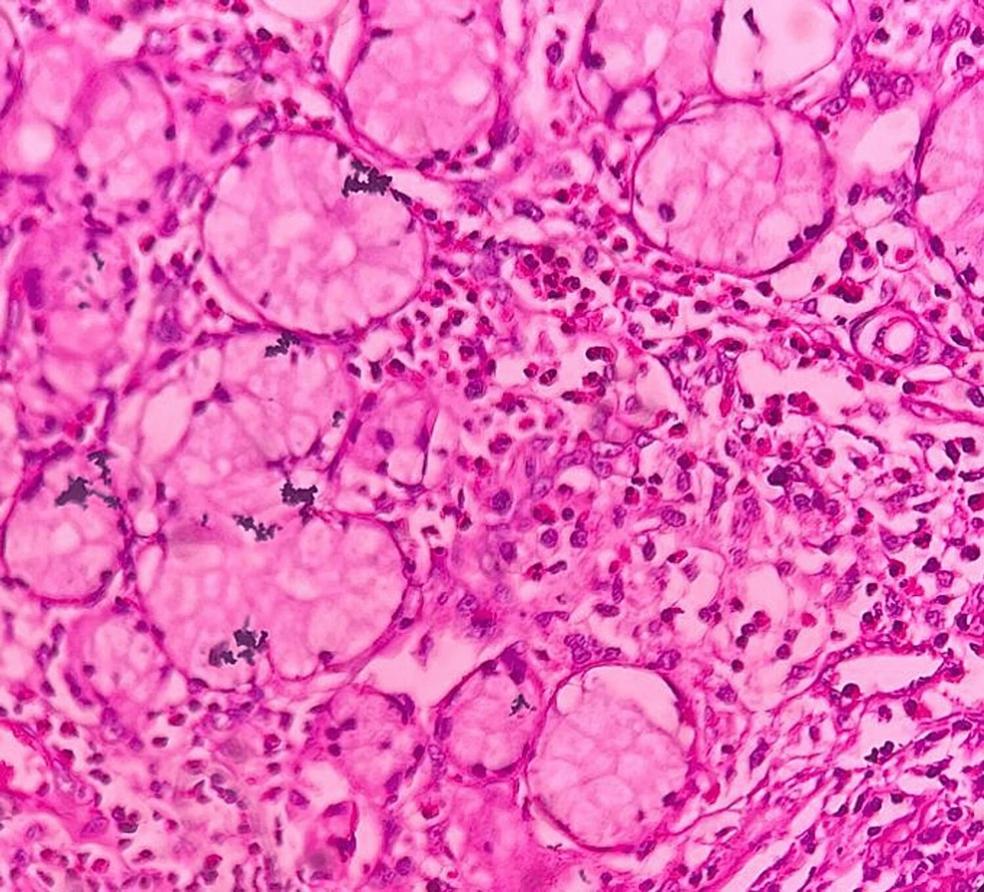
Histology showing features of salivary gland disease (hematoxylin and eosin stain, 100× magnification)

**Figure 9:**
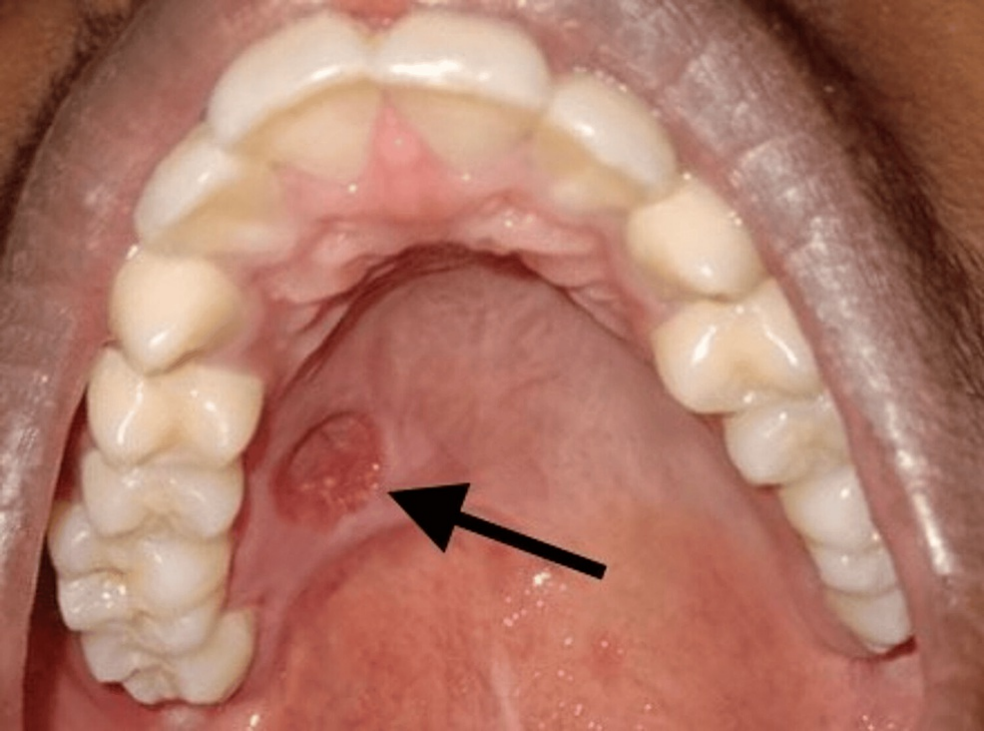
Post-operative follow-up revealing satisfactory wound healing (arrow)

The biopsy revealed salivary gland tissue composed of several lobules of mucous acini, in a background of dense collagenous connective tissue stroma with moderate vascularity. Acinar destruction was evident in several areas accompanied by a marked mixed inflammatory cell infiltrate composed of eosinophils, neutrophils, lymphocytes, and plasma cells, and around the ducts. Additionally, there was evidence of overlying para-keratinized stratified squamous epithelium exhibiting pseudoepitheliomatous hyperplasia in a few areas, suggestive of surface epithelium (Figure 8). Blood investigations showed elevated serum IgE levels (476.30 IU/ml). The histopathological report confirmed the diagnosis of primary hyper-IgE-related salivary gland disease (PHIESD). Post-operative evaluation of the surgical site revealed satisfactory healing with no signs of recurrence during six months of follow-up (Figure 9). 

## Discussion

Inflammatory autoimmune sialadenosis is one of the rarer disorders that have seldom been reported in the literature. This autoimmune inflammation may affect the salivary acini or the salivary ducts. Kimura’s disease [4,5], immunoglobulin G4 (IgG4)-related sialadenitis (IgG4-RS) [6], eosinophilic sialodochitis [7] are considered to be part of hyper-IgE-related salivary gland diseases. Kimura’s disease and IgG4-RS are associated with granulomatous disease involving multiple organs. These diseases present with sialadenosis, eosinophilia, lymphocytic infiltration, and an increase in blood IgE levels [8-10].

However, seldom isolated autoimmune inflammation of salivary glands with increased IgE levels has been reported in the literature without systemic involvement and has been categorized as PHIESD. Precise diagnosis of these lesions is essential in order to optimize the treatment strategies as the systemic involvement by autoimmune inflammation may warrant immune modulators with minimal role for surgical intervention [11].

PHIESD is a rare condition with unique clinicopathological features that warrant comprehensive analysis and summary. As limited information is available in medical literature about this disease, it is crucial to thoroughly examine and understand its characteristics [8]. In terms of demographic characteristics, PHIESD primarily affects young individuals, with a significant proportion of children comprising a substantial portion of the patient population, as observed in the present case. Similar to secondary hyper-IgE-related salivary disorders like IgG4-RS and eosinophilic sialodochitis, the main clinical manifestation was the asymptomatic enlargement of multiple salivary glands [9]. A key distinguishing factor from eosinophilic sialodochitis was the absence of any associated allergic history. Similar to IgG4-RS, it has been postulated that the infiltration of IgE-positive cells and elevated levels of serum IgE might be associated with inherent immune dysregulation at an innate level in PHIESD rather than being an allergic reaction.

The majority of IgE-positive cells in the salivary gland tissues were membrane-based, suggesting that they may largely be immune cells with IgE bound to membrane receptors. The principal IgE receptor is on mast cells, and IL-4 is the primary cytokine that induces IgE synthesis. This explains the sporadic infiltration of IL-4-positive cells and mast cells. The mild degree of the lesions or inactive IgE-related reactions in the local tissues may be to blame for the absence of the allergy-related cytokines, IL-5 and IL-13. IgE production is seen in the peripheral blood and locally in many human tissues (such as the nose and lungs) in individuals with IgE-mediated disease, which is influenced by genetic, racial, immunological, and environmental variables [9-12].

PHIESD presents with an insidious onset in the pediatric age group of 7-14 years with women being more commonly affected. The presence of comorbid allergic condition has been reported to be among 46.7% of patients with hyper-IgE-related salivary gland disease. However, this incidence may be higher in patients with eosinophilic sialodochitis [11]. Hence, a thorough evaluation of the patients’ systemic condition with regular follow-up is essential to rule out the granulomatous involvement of multiple organs. The present case scenario is intriguing as it is one of the rare cases that have been reported in the minor salivary glands.

Management of patients with PHIESD is an area of concern as minimal evidence is available in the literature. Very few reports of PHIESD have been reported in the literature, with most of them being reported bilaterally in major salivary glands with inconclusive evidence on management [13]. The options for the management of these disorders have ranged from wait-and-watch to the use of immunosuppressants and the excision of the lesion. Zhu et al. [8] studied 15 patients with PHISED of major salivary glands and reported no spontaneous regression of the disease, unlike the juvenile parotitis disease entity as reported by Tucci et al. [13]. The anatomical location of the lesion, effects on the function of the stomatognathic system, and systemic involvement seem to primarily dictate the treatment of choice.

Interventions for these lesions, especially in children, often lean towards a conservative approach. The recommended method, with utmost caution, is to adopt a wait-and-watch strategy. Self-care measures such as daily glandular massage, sialagogues, and chewing gum can help alleviate salivary gland distension. Allergen testing, anti-allergic therapy, and avoidance of the allergens may be helpful if known allergens are established. Immune modulator therapy is advisable in patients with multi-system involvement as reported by Baer et al. [14].

Management of isolated minor salivary gland PHIESD has not been reported in the literature so far. Though wait-and-watch appears to be an ideal option considering the pediatric age group, extreme caution on regular follow-up needs to be emphasized in these cases as the probability of low-grade mucoepidermoid carcinoma or adenoid cystic carcinoma cannot be ruled out, especially in the palatal minor salivary glands [15]. Excision may become the treatment of choice when the lesion interferes with functions including mastication and deglutition and proper reconstruction must be planned [16].

The exact nature of the disease, its underlying mechanisms, and the optimal management approach are yet to be fully determined. Existing literature on this condition is scarce, reflecting the limited knowledge available. Recurrence after excision of the lesion has seldom been reported. Van de Veen W et al. in their series had a follow-up between 12 and 68 months post-excision and reported no recurrence, and no regression was noted in unoperated cases. Anti-allergic treatment was associated with recurrence, warranting long-term medical management [11]. However, a long-term diligent follow-up and larger cohort reports are essential in order to have definitive guidelines for the management of these lesions. 

## Conclusions

PHIESD represents a relatively recent pathological entity affecting the salivary glands. Furthermore, the occurrence of this disease specifically in the minor salivary glands of the palate has not been previously documented. In the absence of established guidelines, surgical excision of the affected salivary glands has emerged as an effective treatment option. This approach has shown positive outcomes in managing PHIESD, though further research is needed to ascertain its long-term efficacy and potential alternative therapeutic strategies. Further research endeavors and clinical investigations are warranted to shed light on the optimal management strategies for PHIESD and advance the understanding of this intriguing pathological condition.
